# Digital case-based learning system in school

**DOI:** 10.1371/journal.pone.0187641

**Published:** 2017-11-06

**Authors:** Peipei Gu, Jiayang Guo

**Affiliations:** 1 Software Engineering College, Zhengzhou University of Light Industry, Zhengzhou, Henan, China; 2 School of Electrical and Computer Engineering, University of Cincinnati, Cincinnati, United States of America; Tianjin University, CHINA

## Abstract

With the continuing growth of multi-media learning resources, it is important to offer methods helping learners to explore and acquire relevant learning information effectively. As services that organize multi-media learning materials together to support programming learning, the digital case-based learning system is needed. In order to create a case-oriented e-learning system, this paper concentrates on the digital case study of multi-media resources and learning processes with an integrated framework. An integration of multi-media resources, testing and learning strategies recommendation as the learning unit is proposed in the digital case-based learning framework. The learning mechanism of learning guidance, multi-media materials learning and testing feedback is supported in our project. An improved personalized genetic algorithm which incorporates preference information and usage degree into the crossover and mutation process is proposed to assemble the personalized test sheet for each learner. A learning strategies recommendation solution is proposed to recommend learning strategies for learners to help them to learn. The experiments are conducted to prove that the proposed approaches are capable of constructing personalized sheets and the effectiveness of the framework.

## Introduction

With the rapid development of information technology, e-learning is becoming an inevitable trend of education reform throughout the world [1]. In our knowledge society, the requirements of continuing and lifelong education provide a vast platform for the fast development of e-learning. Because of the flexible learning time, various and abundant learning resources, and distance interaction, e-learning develop rapidly and change the development of education.

There are a variety of learning modes in e-learning environment: computer supported collaborative learning [2–4], personalized learning [5], adaptive learning [6], self-learning [7], distance learning [8], blended learning [9] and web-based learning [10], etc.

The quantity of online multimedia learning resources increases rapidly to fulfill the basic requirements of learning [11]. Along with the big data, machine learning technologies have been applied in learning system [12] [13]. And multimedia systems technologies applied in educational fields is of high interest and have been successfully and widely applied in teaching and learning for educational tools. A musical augmented reality system for children is supported by Rodney et al. It is a useful educational tool, and especially in short and intense interactive learning conditions [14]. Pardo adopts “problem-project-based learning” to meet the dynamic setting in the field of engineering [15]. Hmelo-Silver et al. use video triggers and computer-based technology to facilitate cross-cultural groups in problem-based learning [16]. A system for 3D authoring and presentation in virtual reality environments is proposed by Osawa et al. to help users create 3D educational materials more easily [17]. An educational video compression technique that dynamically allocates the space on the grounds of the importance for each video segment in the educational videos by Mittal et al. [18]. Kulak et al. provides a representative review of case-based learning in science and describes the process of developing case-based learning modules adopted in biochemistry [19]. Krammer et al. conduct the intervention study of video analysis in teacher education to gain the video settings which impacts students’ understanding [20]. Vilsmaier et al propose formats of case-based mutual learning sessions [21].

In our project, we aim at creating a simple environment for inexperienced programmers to build their programming patterns with a stimulating and specific training interface. It integrates multimedia learning resources and offers an easy assistant for learners to test themselves and acquire the guidance in the learning process.

The rest of this paper is organized as follows. Section 2 reviews related work of assembling algorithms for test sheets and approaches of recommending learning strategies in the computing and e-learning environment, respectively. Section 3 describes the framework integrated multi-media resources, learning guidance and testing to support digital case-based learning for variant learners. Section 4 presents our web-based testing algorithm and the solution recommending appropriate learning strategies for learners. Section 5 is the experiments and evaluation. Section 6 draws the conclusion of this paper and presents the future directions.

## Related work

### Algorithms for test sheet construction

The quality of the question bank which is as the candidate set for selecting questions to assemble the test sheet and the algorithm adopted in the construction procedure is the basis of the quality of a test sheet [22]. Many researchers have studied on the test sheet assemble algorithm. Some manually or randomly select test questions from question bank [23]. These approaches are easy, but low efficiency and could not meet the needs of multiple constraints generation situation. Then some researchers concentrate on studying intelligent test sheet generation problem to select an appropriate question set from the questions bank under the condition of multiple requirements [24]. Hwang adopts clustering techniques and dynamic programming to improve the procedure of test sheet with high quality according to specific requirements [24]. Hwang et al. present two improved genetic algorithms to construct test sheet to meet the needs of constraints of specified number of questions and specified range of questions [23]. Lee et al. present an Immune Algorithm to enhance the efficiency of near-optimal test sheet generation [25]. Yin et al. adopt particle swarm optimization (PSO) to improve the efficiency of generating near-optimal serial test sheets from large question bank for meeting multiple assessment requirements in test sheet generation [26].

### Learning strategies recommendation

Appropriate adoption of learning strategies can contribute to the efficiency of learners learning procedure. Some researchers concentrate on recommending learning strategies adaptively in specific areas such as language and mathematics learning areas. Ghinea et al. concentrate on recommending learning strategies according to personal tutoring requirements. Some researchers study on the experimental system in programming learning [27] [28]. Chang et al. develop a programming learning system for beginners with the completion strategy [29]. Paula et al. propose a recommendation system to help students in programming contests [30]. Motivation would aid learners to achieve efficient learning in higher education [31].

### Digital case-based learning framework

In the case-based learning system, it is a problem how to integrate resources as the learning case.

One learning material could be audio, ppt, doc, or video. So it is an important question to help learners learning effectively with multi-media resources. In this section, every learning unit includes three parts: cases, learning strategies and testings. The cases are for learning, learning strategies are for offering an assistant to learners to learn better, and testings can provide learner getting his/her knowledge hierarchy. Learning strategies and testings can make the learning process easier.

**Fig 1:**
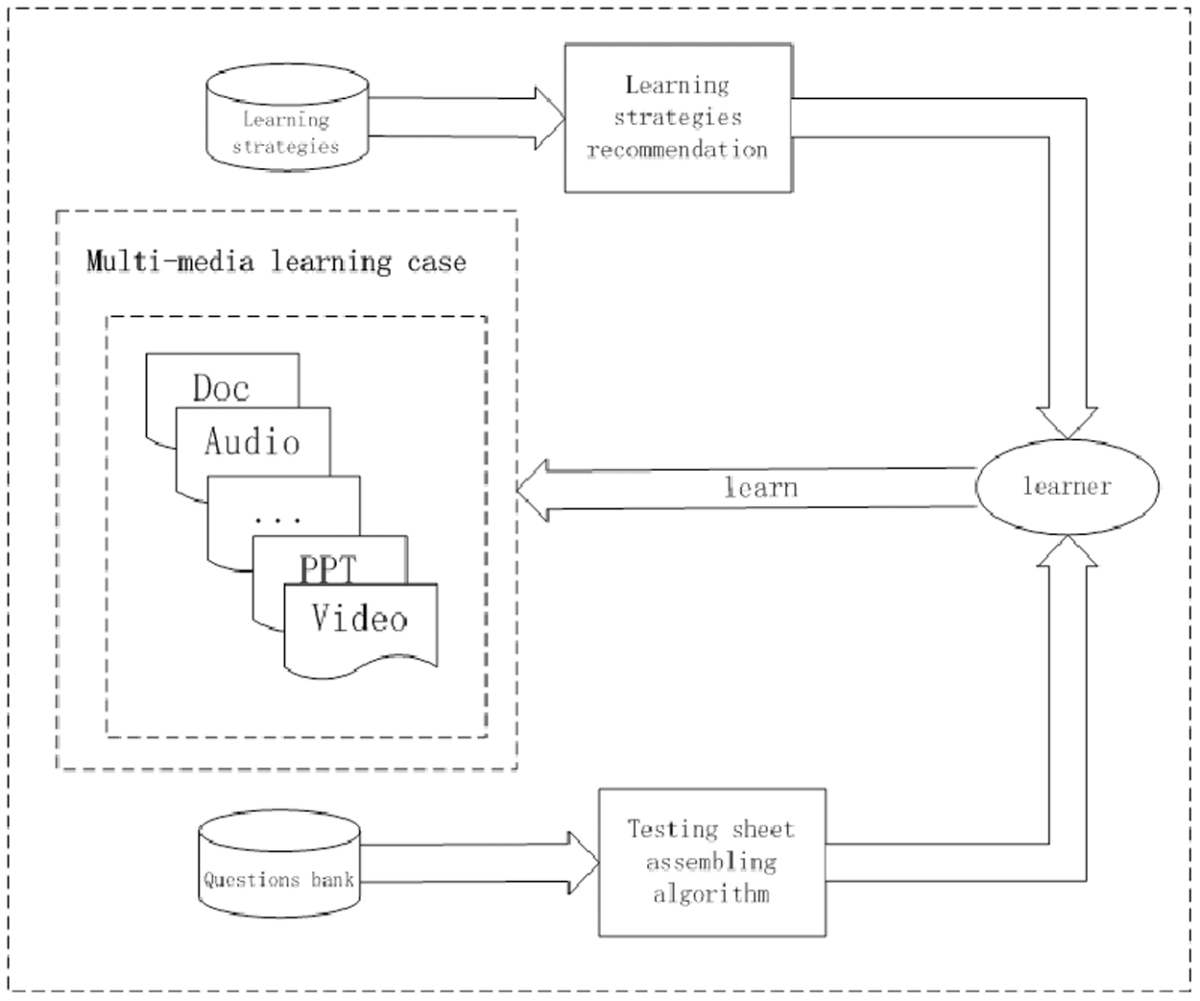
The framework of the digital case-based learning system.

A framework of digital case-based learning is proposed which covers the whole learning process. The proposed framework which is as Fig 1 shown consists of two components: web-based testing and learning strategies recommendation.

In web-based testing, a test sheet is generated according to multiple constraints to satisfy different learner’s evaluation requirements. An improved personalized genetic algorithm to assemble personalized test sheet with more non-mastered questions and more questions not adopted frequently.

In learning strategies recommendation, a solution is proposed to solve the general learning strategies recommendation problem in the digital case-based learning system. It adopts decision tree to adjust the learning strategies recommended set.

### Main technologies

The proposed framework covers learner’s whole learning process. In this section, we emphasize on introducing web-based testing and learning strategies recommendation solution.

### Web-based testing

#### Description of problem

The personalized test sheet generation problem is a multi-objective problem with multi-assessment constraints for student *j*. It concentrates on generating a test sheet which satisfies multiple constraints and levels of mastered knowledge points for student *j* by selecting a certain number of questions from a candidate question set *Qt*_*1*_, *Qt*_*2*_, *…*, *Qt*_*n*_.

There are four relative attributes of each question *Qt*_*i*_: difficulty degree *diff*_*i*, *1≤i≤n*_, discrimination degree *dis*_*i*, *1≤i≤n*_, usage degree *u*_*i*, *1≤i≤n*_, preference information *pref*_*ji*_,_*1≤i≤n*_. The multiple test sheet constraints of the personalized test sheet generation problem include question quantity *q*, expected difficulty degree *diff* and expected discrimination degree *dis*.

We assume that there are *n* questions *Qt*_*1*_, *Qt*_*2*_, *…*, *Qt*_*n*_ in the question bank. *m* knowledge points *Kp*_*1*_, *Kp*_*2*_, *…*, *Kp*_*m*_ are involved in the test. In our test subject, we assume that one knowledge point corresponds to many questions and one question belongs to one knowledge point.

The variables used in describing problem are defined as follows:

1) *x*_*i*, *1≤i≤n*_: *x*_*i*_ = 1 represents that question *Qt*_*i*_ is included in the test, and *x*_*i*_ = 0 otherwise.
2) *q*: question quantity in the final test sheet.
3) *dis*_*i*, *1≤i≤n*_: degree of the discrimination of *Qt*_*i*_.
4) *diff*_*i*, *1≤i≤n*_: degree of the difficulty of *Qt*_*i*_.
5) *u*_*i*, *1≤i≤n*_: degree of the usage of *Qt*_*i*_.
6) *dis*: the expected discrimination degree of the whole test.
7) *diff*: the expected difficulty degree of the whole test sheet.
8) *pref*_*ji*_,_*1≤i≤n*_: the preference information in *Qt*_*i*_ of student *j*, *pref*_*ji*_ = 0 represents that student *j* has mastered question *Qt*_*i*_, and *pref*_*ji*_ = 1 otherwise (detailed calculation equation is shown as (4)).
9) *af%*: expected percentage of level of non-mastered concepts of a subject in the test.
10) *Z(pref*_*j1*,_ *pref*_*j2*, *…*,_ *pref*_*ji*, *…*,_ *pref*_*jn*_*)*: preference information of student *j*.

The objective function can be defined as follows:

$$\text{Min}\;f=\frac{\left(\sum_{i=1}^{n}\left|diff_{i}-diff\right|\;x_{i}+\sum_{i=1}^{n}\left|dis_{i}-dis\right|\;x_{i}\right)}{(2*\sum_{i=1}^{n}x_{i})}-Z(pref_{j1},\ldots pref_{ji},\ldots pref_{jn})$$ (1)

where

$$Z(pref_{j1},pref_{j2}\ldots pref_{ji},\ldots pref_{jn})=\{\begin{array}{c} \;0.5\;,\;\sum_{i=1}^{n}pref_{ji}x_{i}>=q*af\%\\ \;-0.5\;,\;\sum_{i=1}^{n}pref_{ji}x_{i}<q*af\% \end{array}$$ (2)

In (1), variable *x*_*i*_ represents question *Qt*_*i*_ is chosen or not in the final test sheet. In (2), *pref*_*ji*_ represents student *j* has mastered question *Qt*_*i*_ or not, *∑*^*n*^_*i = 1*_ *pref*_*ji*_ *x*_*i*_ represents the quantity of questions on the test sheet that are mastered by student *j*, and q*af% indicates the expected quantity of non-mastered questions that should be selected in the final test sheet. The preference information *Z(pref*_*j1*, *…*,_ *pref*_*ji*, *…*,_ *pref*_*jn*_*)* makes for narrowing the gap between final difficulty and discrimination degree and expected difficulty and discrimination degree.

#### Improved personalized genetic algorithm

In the previous work, to solve the personalized test sheet generating problem, we propose a personalized genetic algorithm named PGA which is an improved GA that incorporates students’ preference information into crossover operator [32]. And based on this, incorporated with usage degree, we improve the former algorithm in crossover and mutation process to solve this problem effectively.

Preference information of student can help us better acquiring the learning level of the students. And the frequency of usage for questions can help constructing more effective test sheet. Improved personalized genetic algorithm (IPGA) can assemble test sheet satisfied students personal requirements.

In this paper, *pref*_*jkps*, *1≤s≤m*_, the level of mastered knowledge point *kp*_*s*_ for student *j* and usage degree *u*_*i*_ are incorporated into the assembling process for constructing a personalized test sheet for student *j*. The *pref*_*jkps*, *1≤s≤m*_ is defined as follows:

$$pref_{jkp_{s}}{,}_{1\leq s\leq m}=\{\begin{array}{c} 0\;,YNum>=aNum*pf\%\\ 1\;,YNum<aNum*pf\% \end{array}.$$ (3)

In (3), variable *YNum* represents the right quantity of answering the questions which correspond to knowledge point *kp*_*s*_ for student *j*. Variable *aNum* represents the quantity of answering the questions which correspond to knowledge point *kp*_*s*_ for student *j*. Variable *pf%* is the description of mastered level of knowledge point *kp*_*s*_ in answering right for student *j*. Different levels can be assigned based on the actual needs of the situations. For example, when more than *pf%* of questions are answered right, the mastered level of knowledge point *kp*_*s*_ is 0 (0 indicates student *j* has mastered the knowledge point *kp*_*s*_, 1 is not).

The *pref*_*ji*_,_*1≤i≤n*_ for question *Qt*_*i*_ which corresponds to knowledge point *kp*_*s*_ is as follows:

$$pref_{ji},{\;}_{1\leq i\leq n}=\{\begin{array}{c} 0\;,\;\;\;\;\;if\;student\;j\;answers\;\;Qt_{i}\;is\;right\\ \;1\;,\;\;\;\;\;\;if\;student\;j\;answers\;\;Qt_{i}\;is\;wrong\\ pref_{jkp_{s}}\;,\;\;\;if\;student\;j\;have\;not\;ansewered\;\;Qt_{i}\; \end{array}.$$ (4)

The usage degree of question *Qt*_*i*_ indicates the selected frequency in the former constructed test sheets. When students take an examination, some questions may be selected by teachers many times, so usage degree of question is incorporated into the construction procedure would be useful for the examination.

The definition of *u*_*i*_ is defined as follows:

$$u_{i}=0.8^{g}$$ (5)

The initial value of *u*_*i*_ is 1. *g* is the frequency of occurrence of question *Qt*_*i*_. Together with question *Qt*_*i*_ is selected in the final test sheet, the value of *u*_*i*_ is cut down. The lower *u*_*i*_ is, the more frequent question *Qt*_*i*_ has been selected in the final test sheet.

**Fig 2:**
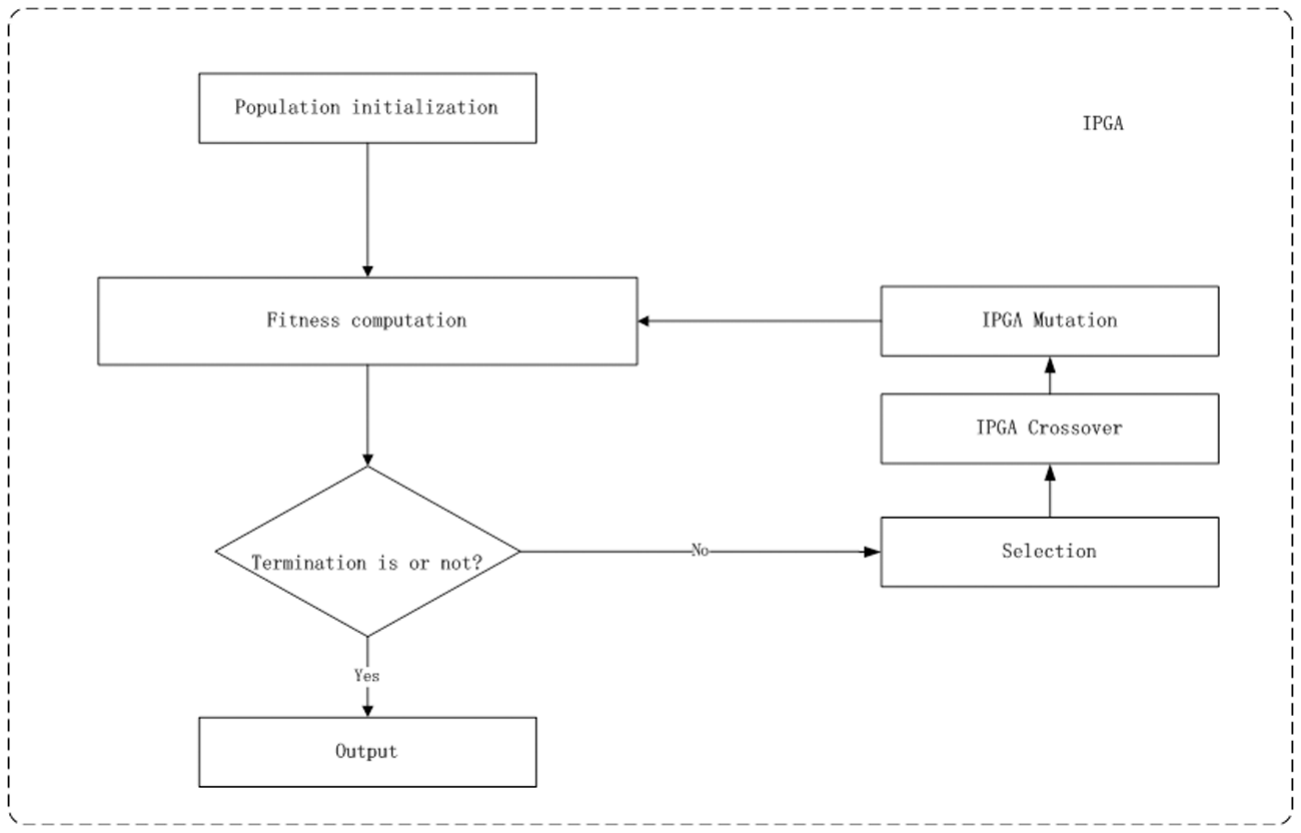
IPGA procedure.

IPGA process is based on the traditional GA. The procedure of IPGA is as Fig 2 shows.

The description of the IPGA procedure is as follows:

1) Initialize the population.
2) Compute fitness with (1).
3) Go to step *8)* if the termination criterion is satisfied.
4) Select mother individual *ind*_*mum*_ and father individual *ind*_*dad*_ according to roulette algorithm.
5) Crossover *ind*_*mum*_ and *ind*_*dad*._ (IPGA crossover procedure).
6) Mutate (IPGA mutation procedure).
7) Go to *2)*.
8) The best generation is the final test sheet.

**Fig 3:**
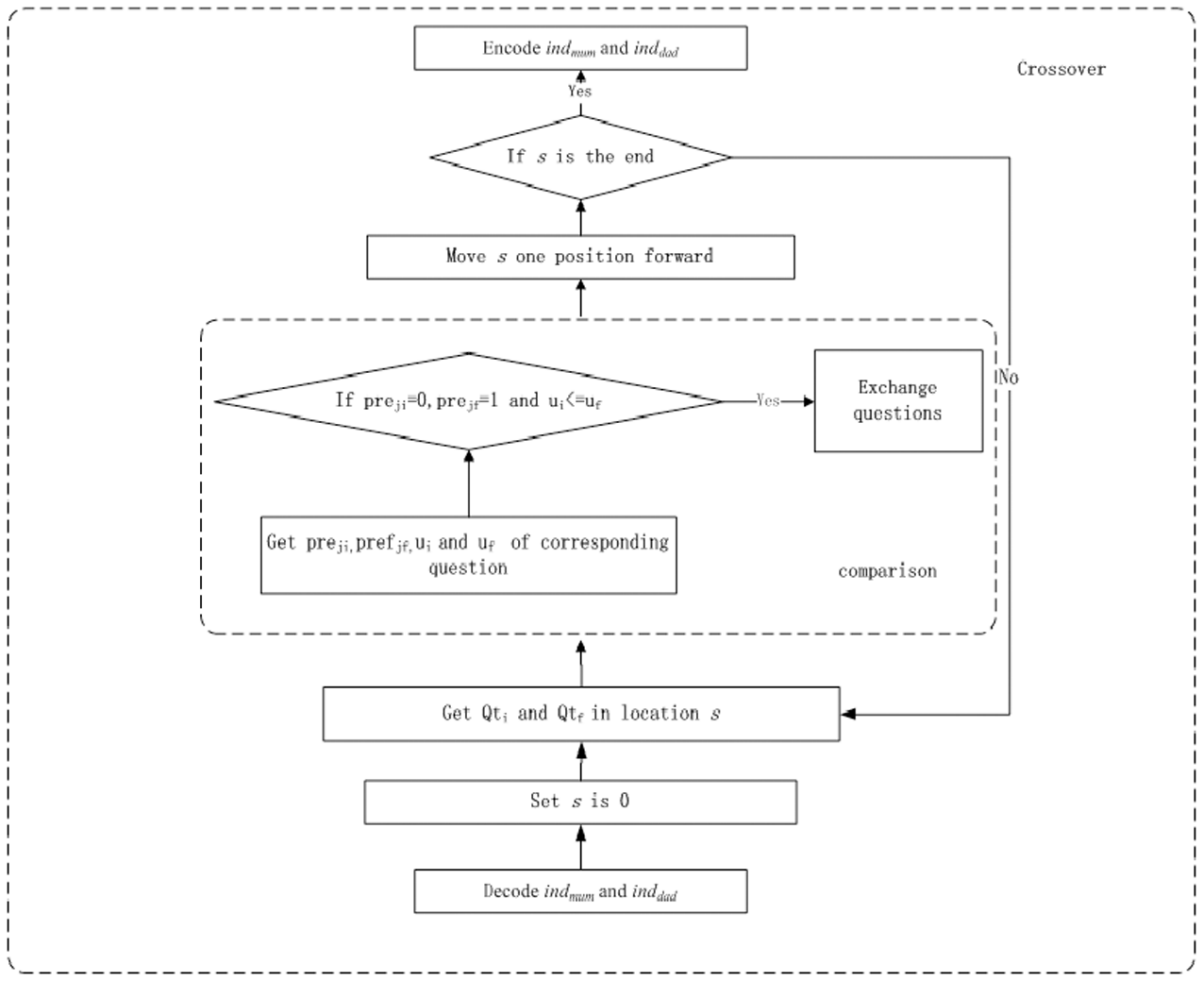
IPGA crossover procedure.

IPGA incorporates preference information and usage degree into the crossover process as Fig 3 shows.

The description of the IPGA crossover procedure is as follows:

1) Decode mother individual *ind*_*mum*_ and father individual *ind*_*dad*._
2) Set the crossover location *s* is 0.
3) Get *Qt*_*i*_ and *Qt*_*f*_ in the corresponding location *s*.
4) Get *pre*_*ji*_ and *u*_*i*_ of question *Qt*_*i*_, and get *pref*_*jf*_ and *u*_*f*_ of question *Qt*_*f*_.
5) Compare *pre*_*ji*_ and *pre*_*jf*_, and *u*_*i*_ and *u*_*f*_, if *pre*_*ji*_ = 0, *pre*_*jf*_ = 1 and *u*_*i*_< = *u*_*f*_ then exchange question *Qt*_*i*_ and *Qt*_*f*_.
6) *s* plus one. Move crossover location *s* forward one position.
7) If *s* is the end of *ind*_*mum*_, encode *ind*_*mum*_ and *ind*_*dad*,_ and then go to IPGA mutation procedure.
8) Go to 3).

**Fig 4:**
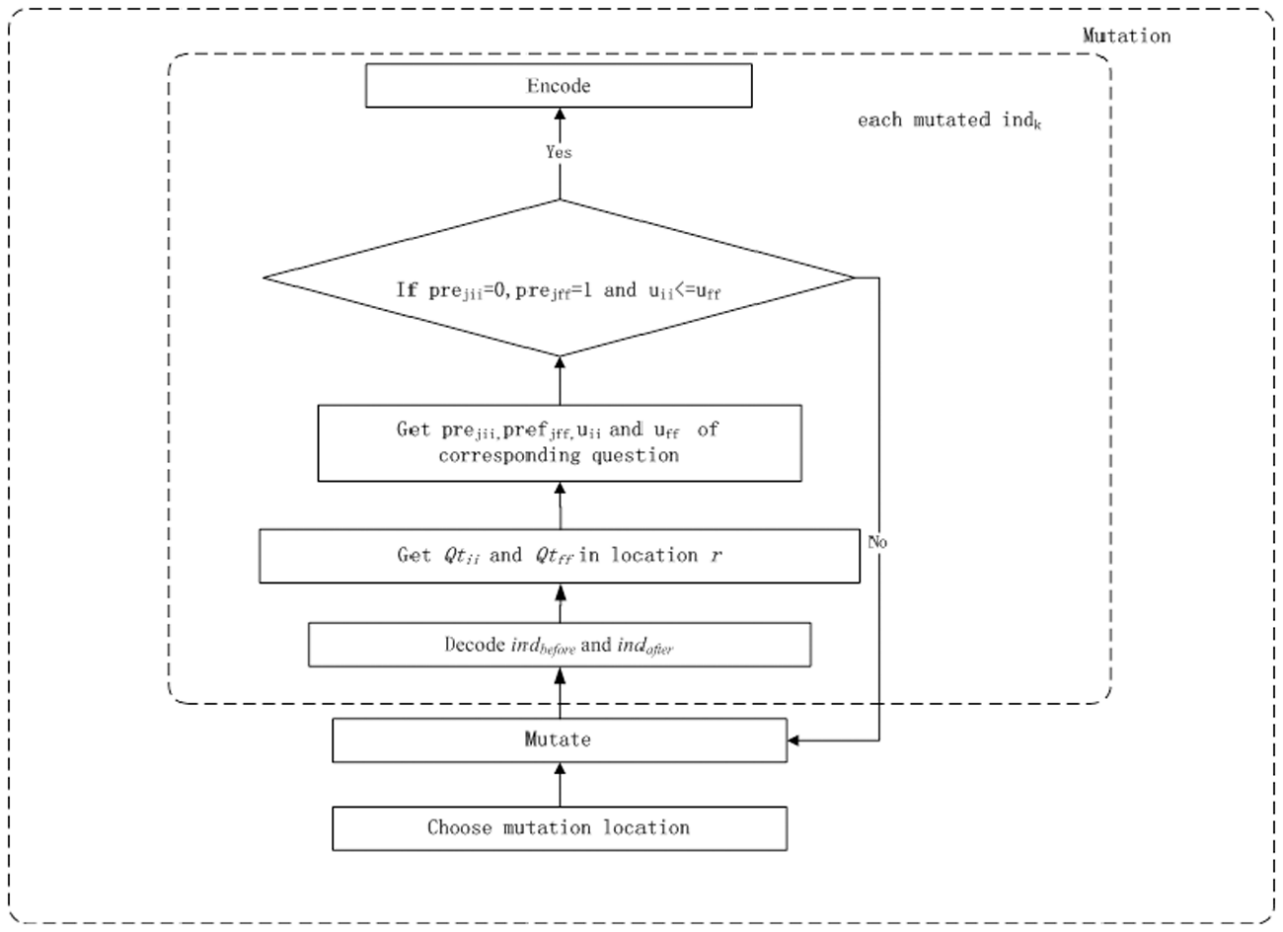
IPGA mutation procedure.

IPGA incorporates preference information and usage degree into the mutation process as Fig 4 shows.

The IPGA mutation procedure can be described as follows:

1) Choose mutation position randomly.
2) Mutation.
3) For each mutated *ind*_*k*_.
  3.1) Decode old individual *ind*_*before*_ and mutated individual *ind*_*after*_.
  3.2) Get *Qt*_*ii*_ and *Qt*_*ff*_ in the corresponding location *r*.
  3.3) Get *pre*_*jii*_ and *u*_*ii*_ of question *Qt*_*ii*_, and get *pref*_*jff*_ and *u*_*ff*_ of question *Qt*_*ff*_.
  3.4) Compare *pre*_*jii*_ and *pref*_*jff*_, and *u*_*ii*_ and *u*_*ff*_, if the terminal condition (*pre*_*jii*_ = 0, *pref*_*jff*_ = 1 and *u*_*ii*_< = *u*_*ff*_) is not satisfied then go to 2).
  3.5) Encode.

### Learning strategies recommendation

In our previous work, we propose a learning strategies recommendation approach for the e-learning system which integrated with multiple learning sources, such as video, documents or some other teaching materials [33]. Based on the former general learning strategy recommendation algorithm, we propose a learning strategy recommendation solution for our digital case-based learning system.

#### Learning strategies

Scarcella & Oxford have researched the learning strategies and proposed the definition of learning strategies as “specific actions, behaviors, steps, or techniques—such as seeking out conventional partners or giving oneself encouragement to tackle a difficult language task—used by learners to enhance their own learning” [34].

In the digital case-based learning system, it is important to recommend learning strategies for learners for the learning strategies can help improve the ability effectively in using the multiple learning video resources.

In our previous work, we proposed a learner model to cover the various properties of learners for making the final learning strategies recommend list. We should collect five parts five parts of information: static properties, dynamic properties, affective information, history of learning strategy choosing, and test results of all tests [33] [35].

Five groups of learning strategies are adopted in our digital system:

1) Meta-cognitive strategies: It contains plan making and summarization of data. These learning strategies can support certain approaches to help learners studying prospective or summarize the previous knowledge.
2) Memory-related strategies: It contains learning and reviewing. These strategies can support a number of methods to improve the whole learning procedure.
3) Compensatory strategies: It contains some compensatory methods to avoid forgetting knowledge. In the normal learning process of acquiring knowledge, there must be some knowledge point we omitted or forgotten. These strategies can support some approaches to release early and iterate.
4) Affective strategies: It contains mental strategies, such as rewards or encouraging mechanism. These strategies can support certain approaches to re-build confidence and motivation of learners, attract learners’ imagination.
5) Social strategies: It contains the ask and help from the community, friends, experts and other individual learners. It represents the openness of the digital system.

#### A learning strategy recommendation solution

**Fig 5:**
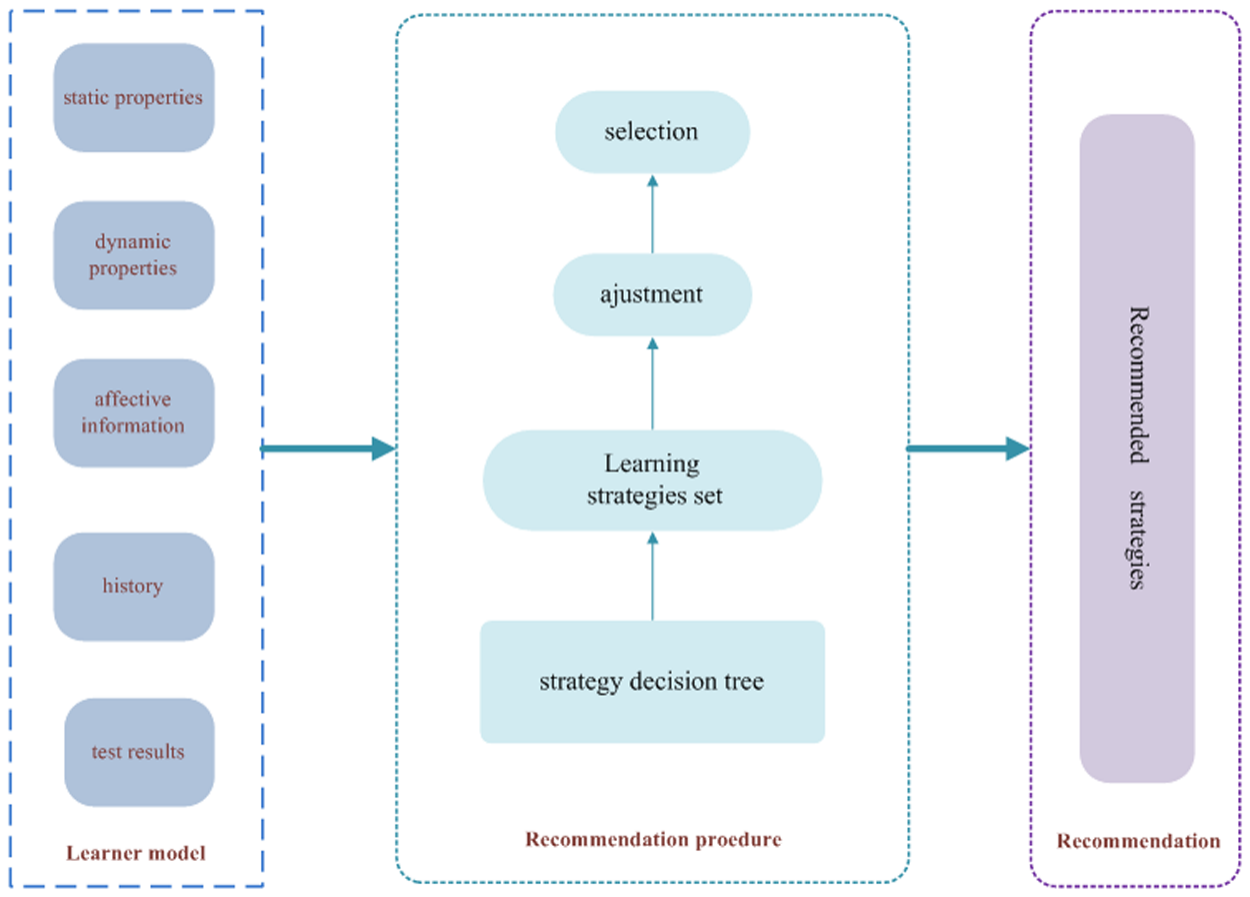
Learning strategy recommendation solution for the second and third type of learner.

A learning strategy recommendation solution for recommending proper learning strategies is proposed which includes two aspects: modeling learners, recommendation procedure as Fig 5 shows.

There are three types of learners in the digital case-based learning system. 1) Unregistered learners or registered learners who are the newbies. 2) Registered learners who have no idea about the learning strategies recommendation solution or have no interest in the recommended learning strategies. 3) Registered learners who are familiar with the recommended solution and prefer adopting the learning strategies recommended.

For the first type of learner, we recommend top 2 from the learning strategies recommendation history of all learners ranking according to the calculation of (6).

Learning strategies recommendation process for the second and third type of learner can be described as follows:

1) Select learning strategy based on the learning strategies decision tree.
2) Add learning strategy as the candidate in learning strategies recommendation set.
3) Adjust the candidate learning strategies recommendation set.
4) Select top 2 from the candidate learning strategies recommendation set as the final recommendation set to offer.

A learning strategies decision tree is constructed based on C4.5 [36] (see S1 File).

In the solution, when learning strategies set is selected based on the learning strategies decision tree, we should execute adjustment on the set. The adjustment can be described as follows:

1) Select the favorite top 3 learning strategy.
2) Add them into the learning strategies candidate set.
3) Calculate the influence factor according to (6).
4) Rank learning strategies in the set based on the calculation of the influence factor *if*.

We assume *r* is the frequency of occurrence of one learning strategy in the learning strategies adoption history. *m* is the size of learning strategies in adoption history. *e* is the recommended weight. If the learning strategy is recommended based on the learning strategies decision tree, *e* is 1, else *e* is 0.8 for the reason of balancing the learning strategy from the decision tree and the history.

The calculation rules of influence factor *if* is as follows:

$$if=e*\frac{r}{m}$$ (6)

## Experiments and evaluation

### Web-based testing

To evaluate the performance of the proposed IPGA, a series of experiments are conducted by comparing it with Traditional GA in three aspects: execution time, usage degree, final result quality, and final result distribution between mastered and non-mastered questions.

The simulation experiments are conducted for constructing final test sheet which contains 20 questions of the best difficulty and discrimination degree with applying the algorithms 10 times. All algorithms used in the experiments are coded in Java Language conducted on a personal computer with Intel (R) Core (TM) 2 Duo CPU @ 2.53GHz and 1.93GB memory.

There are all the parameters of 10000 simulation questions which refer to 121 knowledge points in our candidate testing bank (see S2 File). There are 5 degrees of the difficulty and discrimination in the test sheet assembling procedure as described in Table 1.

**Table 1:** Difficulty and discrimination degree classification.

Degree	Lowest	Lower	Normal	Higher	Highest
**Value**	1.0	2.0	3.0	4.0	5.0

Value 1.0~5.0 are adopted to represent the lowest~the highest difficulty and discrimination degree respectively. And *af%* which is the expected percentage of non-mastered questions that should be selected in the final test sheet is set to 60%.

There are 3 learners in the digital case-based learning system involving in these experiments, and they are Zhao, Qian and Sun. The level of mastered knowledge point is as described in Table 2. The percentage value is high means the better the learner get the corresponding knowledge point. Otherwise, the low percentage value indicates low learning level referring to the knowledge point. For example, the mastery degree of Qian is higher than Zhao and This would impact on the test sheet assemble procedure.

**Table 2:** Percentage of mastered knowledge point referring to different learners.

Learners	Zhao	Qian	Sun
**Percentage**	36.4%	58.7%	67.8%

The experiments results of the difference of final average difficulty with expected difficulty in applying with IPGA and Traditional GA for 3 learners in 10 times are shown in Table 3. The experiments results of the difference of final average discrimination with expected discrimination in applying with IPGA and Traditional GA for 3 learners in 10 times are shown in Table 4. The expected difficulty and discrimination degree of (*diff*, *dis)* are *(1*.*0*, *1*.*0)*, *(2*.*0*, *2*.*0)*, *(3*.*0*, *3*.*0)*, *(4*.*0*, *4*.*0) and (5*.*0*, *5*.*0)*. The difference of (Diff-dif) and (Dis-dis) between IPGA and Traditional GA are negative which indicates IPGA gets small gap between final average degrees and expected degrees than Traditional GA and it means we could achieve better final difficulty and discrimination degree in applying with IPGA. As Tables 3 and 4 show, there are 17 negative values which mean we can get better final difficulty and discrimination degree in applying with IPGA 17 times and Traditional GA 13 times. So we achieve better experiment results with IPGA than Traditional GA in difficulty and discrimination degree.

**Table 3:** The difference of final average difficulty with expected difficulty between IPGA and Traditional GA.

*(diff*, *dis)*	Difference of (Diff-diff) between IPGA and Traditional GA		
	Zhao	Qian	Sun
**(1.0, 1.0)**	-0.0015	0.019	0.0155
**(2.0, 2.0)**	0.0055	0.0035	0.032
**(3.0, 3.0)**	-0.003	0.0075	0.0055
**(4.0, 4.0)**	-0.0035	0.01	-0.0195
**(5.0, 5.0)**	-0.018	-0.009	-0.1036

Diff is the final average difficulty degree in assembling 10 times procedure. diff is the expected difficulty degree.

**Table 4:** The difference of final average discrimination with expected discrimination between IPGA and Traditional GA.

*(diff*, *dis)*	Difference of (Dis-dis) between IPGA and Traditional GA		
	Zhao	Qian	Sun
**(1.0, 1.0)**	0.009	0.0165	0.0335
**(2.0, 2.0)**	-0.006	0.0235	0.0295
**(3.0, 3.0)**	-0.0335	-0.0205	-0.019
**(4.0, 4.0)**	-0.015	-0.0445	-0.0195
**(5.0, 5.0)**	-0.0085	-0.0405	-0.0185

Dis is the final average discrimination degree in assembling 10 times procedure. dis is the expected discrimination degree.

The total quantities of non-mastered questions by applying IPGA and Traditional GA for learner Zhao, Qian and Sun are shown in Table 5. The difference of total quantities of non-mastered questions between IPGA and Traditional GA for 10 times are positive which indicates the quantity of non-mastered questions in applying with IPGA are more than Traditional GA and it means we could achieve better final non-mastered questions in applying with IPGA. When assembling the test sheets, IPGA can get more non-mastered questions in final test sheet. IPGA achieves good non-mastered questions distribution than traditional GA.

**Table 5:** Experiment results of the difference of total quantities of non-mastered questions between IPGA and Traditional GA in applying 10 times on the best test sheet construction for 3 learners.

*(diff*, *dis)*	Difference of the total quantities of non mastered questions between IPGA and Traditional GA in 10 times		
	Zhao	Qian	Sun
**(1.0, 1.0)**	6	41	43
**(2.0, 2.0)**	31	25	42
**(3.0, 3.0)**	23	39	30
**(4.0, 4.0)**	13	27	23
**(5.0, 5.0)**	10	29	41

The average usage degree by applying IPGA and Traditional GA for learner Zhao, Qian and Sun are shown in Table 6. The difference of average usage degree between IPGA and Traditional GA are positive which indicates we can get high usage degree in applying with IPGA. As Table 6 shows, there are 9 positive values which mean we can get final test sheet of higher usage degree 9 times with IPGA and Traditional GA 6 times. So we achieve more questions of low frequency with IPGA than Traditional GA.

**Table 6:** Experiment results of difference of average usage degree between IPGA and Traditional GA.

*(diff*, *dis)*	Difference of the average usage degree between IPGA and Traditional GA		
	Zhao	Qian	Sun
**(1.0, 1.0)**	0.00965	0.00565	0.01005
**(2.0, 2.0)**	-0.00075	-0.00025	0.00455
**(3.0, 3.0)**	-0.00665	0.00715	-0.0021
**(4.0, 4.0)**	-0.0141	0.00565	0.0035
**(5.0, 5.0)**	-0.00325	0.00105	0.01935

**Fig 6:**
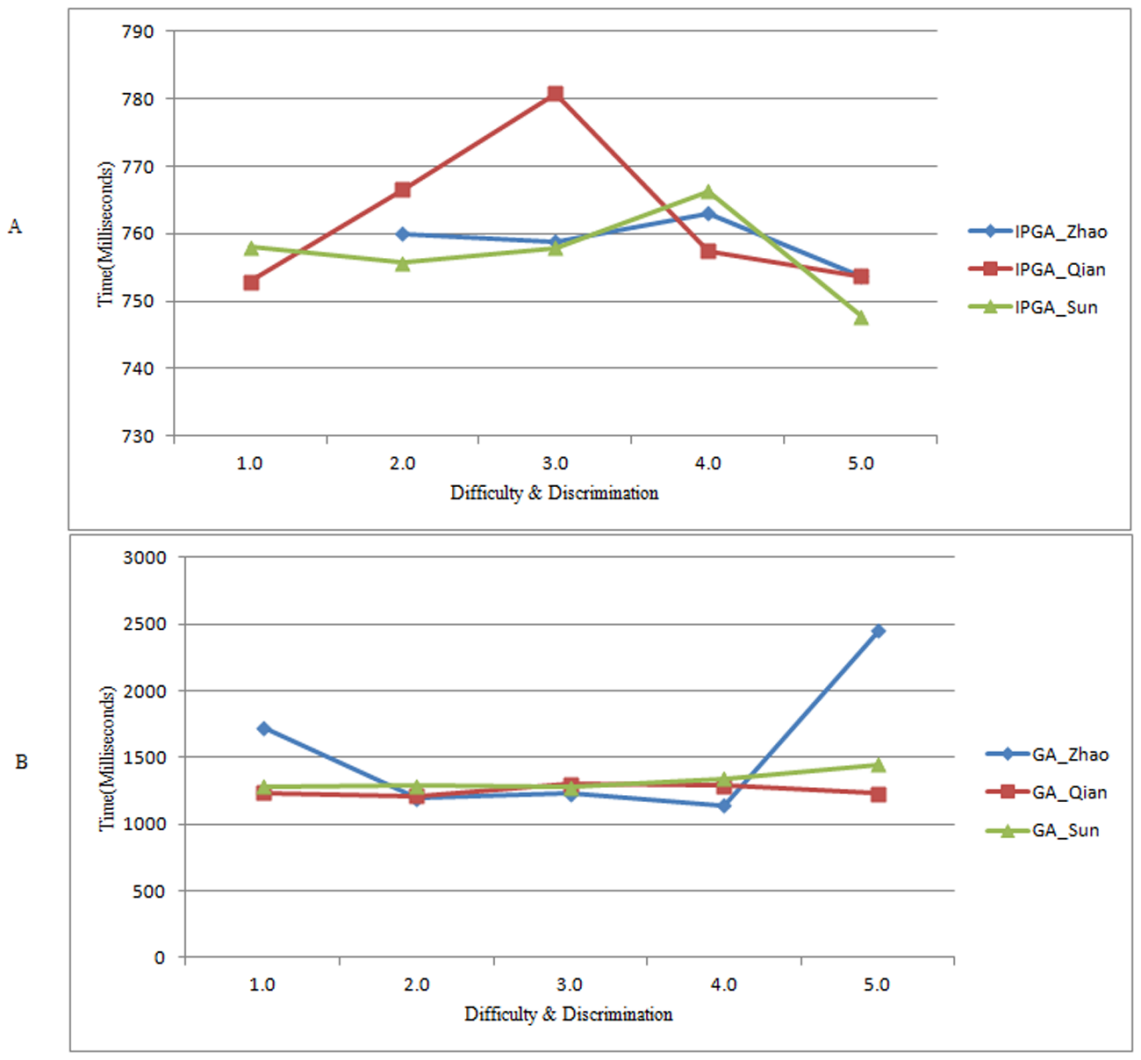
Average execution time in different difficulty and discrimination degrees for each learner. Average execution time in applying with IPGA (A). Average execution time in applying with Traditional GA (B).

Fig 6 presents the execution time of IPGA and Traditional GA. There is unusual 12806.9 milliseconds when applying with IPGA for learner Zhao in *(1*.*0*, *1*.*0)* because of its questions distribution, so we remove the value from the final figure. As shown, IPGA consumes shorter time to construct test sheet satisfying multiple constraints for learners in most times. Improved crossover and mutation procedure are proved to be effective for optimization.

### Learning strategies recommendation

In our project, final learning strategies list for learners are selected based on the learning strategies recommendation solution.

If a learner accesses system as a newbie, top 2 learning strategies are recommended according to the learners learning strategies adopting history. For the other registered learners, top 2 learning strategies are recommended based on learning strategies decision tree and adoption history. It can save time and energy for learners when only top 2 learning strategies recommended to them. For example, top 2 learning strategies of learner Zhao are video learning review and community help, so relevant video resources and specific information such as posts, experience, etc, are provided according to the learning strategies. The learner can access relevant learning materials on the home page.

When learners choose to learn video case, multiple assisted learning approaches are provided. Learners can comment, ask or take notes when learning the video case. And when learners finish the test, it marks automatically and rewards learners credits to attract and encourage learners.

## Conclusion

This paper discussed and analyzed kinds of situation which constructing a case-based learning system of multimedia resources. Integrated with learning strategies recommendation and learning testing, the case-based learning system provides the personalized testing and appropriate programming learning strategies for learners.

In our experiments, IPGA algorithm could select availably personalized test sheet for the individual learner. Programming learning strategies recommended to learner achieved good evaluation from the learners. And the case-based learning system can effectively provide a whole learning procedure for the different learner.

In our future work, we will study on the integration and usage approaches of multimedia resources to exert the functionality of digital case-based learning system.

## Supporting information

S1 File LearningStrategiesDecisionTree. (DOC)

S2 File SimulatedDataSet. (XLS)

S3 File AssemblingResult. (XLS)
