# Supplementary material for: Digital case-based learning system in school
Source: PLoS One. 2017 Nov 6;12(11):e0187641. doi: 10.1371/journal.pone.0187641 (PMC5673172; doi:10.1371/journal.pone.0187641)
Supplement: S1 File — (DOC) [file pone.0187641.s001.doc]

**Fig. 1**  **The generated learning strategy decision tree**
